# Phage strategies facilitate bacterial coexistence under environmental variability

**DOI:** 10.7717/peerj.12194

**Published:** 2021-11-04

**Authors:** Esther Voigt, Björn C. Rall, Antonis Chatzinotas, Ulrich Brose, Benjamin Rosenbaum

**Affiliations:** 1 German Centre for Integrative Biodiversity Research (iDiv), Leipzig, Germany; 2 Institute of Biodiversity, Friedrich Schiller University Jena, Jena, Germany; 3 Department of Environmental Microbiology, Helmholtz Centre for Environmental Research-UFZ, Leipzig, Germany; 4 Institute of Biology, Leipzig University, Leipzig, Germany

**Keywords:** Bacteria phage interactions, Virus, Temperate phages, Population dynamics, Biodiversity, Microbial ecology, Resource fluctuations, Environmental variability, Ordinary differential equations (ODEs), Non-linear dynamics

## Abstract

Bacterial communities are often exposed to temporal variations in resource availability, which exceed bacterial generation times and thereby affect bacterial coexistence. Bacterial population dynamics are also shaped by bacteriophages, which are a main cause of bacterial mortality. Several strategies are proposed in the literature to describe infections by phages, such as “Killing the Winner”, “Piggyback the loser” (PtL) or “Piggyback the Winner” (PtW). The two temperate phage strategies PtL and PtW are defined by a change from lytic to lysogenic infection when the host density changes, from high to low or from low to high, respectively. To date, the occurrence of different phage strategies and their response to environmental variability is poorly understood. In our study, we developed a microbial trophic network model using ordinary differential equations (ODEs) and performed ‘*in silico*’ experiments. To model the switch from the lysogenic to the lytic cycle, we modified the lysis rate of infected bacteria and their growth was turned on or off using a density-dependent switching point. We addressed whether and how the different phage strategies facilitate bacteria coexistence competing for limiting resources. We also studied the impact of a fluctuating resource inflow to evaluate the response of the different phage strategies to environmental variability. Our results show that the viral shunt (*i.e*. nutrient release after bacterial lysis) leads to an enrichment of the system. This enrichment enables bacterial coexistence at lower resource concentrations. We were able to show that an established, purely lytic model leads to stable bacterial coexistence despite fluctuating resources. Both temperate phage models differ in their coexistence patterns. The model of PtW yields stable bacterial coexistence at a limited range of resource supply and is most sensitive to resource fluctuations. Interestingly, the purely lytic phage strategy and PtW both result in stable bacteria coexistence at oligotrophic conditions. The PtL model facilitates stable bacterial coexistence over a large range of stable and fluctuating resource inflow. An increase in bacterial growth rate results in a higher resilience to resource variability for the PtL and the lytic infection model. We propose that both temperate phage strategies represent different mechanisms of phages coping with environmental variability. Our study demonstrates how phage strategies can maintain bacterial coexistence in constant and fluctuating environments.

## Introduction

### Theory in bacteria population dynamics

Bacteria are the most abundant and diverse organisms on earth (Whitman, Coleman & Wiebe, 1998; Godfray, Knapp & Oren, 2004). They occur in all habitats from the atmosphere down to the deep oceanic subsurface (Flemming & Wuertz, 2019) and are adapted to a variety of sometimes extreme conditions of temperature, pH, pollution, or salinity, including substantial temporal fluctuations in these parameters. As one of the main drivers of nutrient cycles, they are of great biological importance (Lladó, López-Mondéjar & Baldrian, 2017). In natural food webs, bacteria are basal species that establish an important connection between inorganic resources or dead organic matter and higher trophic organisms (Steffan et al., 2017; Steffan & Dharampal, 2019). However, the ever present question of “Why are there so many bacteria species and how can they coexist despite competing for a limited number of resources?” remains unanswered. This situation is similar to the “paradox of the plankton”: the observed diversity of plankton species is very high despite the fact that the amount of resources restricts the number of competitors (Hutchinson, 1961; Tilman, 1977, 1982). According to classical niche theory, coexisting species should occupy different niches along gradients in resources or environmental conditions to avoid competitive exclusion. Hence, species can coexist if their niches are of limited similarity. This concept of coexistence is difficult to apply to bacterial species, since many bacteria share the same resources and living conditions. The lingering question consequently is: “How can hundreds of bacterial strains coexist on one to few resources?” Some processes such as disturbances (Connell, 1978) and chaotic dynamics (Huisman & Weissing, 1999) can prevent the exclusion of species by relieving competitive pressure and thereby increasing biodiversity on a few resources. Alternatively, top-down control by keystone consumers (Paine, 1980; Leibold, 1996; Power et al., 1996; Jochum et al., 2012) or complex food webs (Brose, 2008; Schneider et al., 2016; Wang & Brose, 2018) can prevent resource-driven competition and extinction. In microbial communities, top-down control can be strongly driven by infection processes that are the main cause of bacterial death and control of population dynamics (Fuhrman, 1999; Noble, Middelboe & Fuhrman, 1999; Weinbauer et al., 2009). Hereby, viruses exhibit a profound influence on the abundance and diversity of bacteria.

### Control of bacterial population dynamics

Bacteriophages (phages) are viruses that exclusively infect bacteria. Their abundance and distribution are linked to their hosts (Clokie et al., 2011). Phages can promote bacterial diversity by acting as a top-down control for highly abundant species (Thingstad, 2000; Ram, Keshri & Sime-Ngando, 2020; Zheng et al., 2021). Furthermore, phages can influence their host by gene transfer (Day et al., 2017) or reprogramming of the host’s gene expression and metabolism (Long, Patterson & Paul, 2007; Hurwitz & U’Ren, 2016). Therefore, phages are of particular interest regarding their relevance in ecosystem functioning (Suttle, 2005). Similar to consumer-resource pairs, bacteria and phages mutually affect each other’s population dynamics (Brockhurst et al., 2007; Matteson et al., 2012; Hiltunen et al., 2017). Bacterial hosts control phage production indirectly by their physiology, such as growth rate, which is in turn often linked to the availability of resources (Zimmerman et al., 2020). Phages employ different strategies or even switch between them. Lytic phages infect and replicate inside their hosts, ultimately killing them to spread (lytic infection). Temperate phages insert their DNA or RNA into the host genome to be replicated with the host (lysogenic infection), but once they get activated they enter the lytic cycle (Howard-Varona et al., 2017). In addition to phages, cell-debris is released during host lysis, which is known as the viral shunt (Wilhelm & Suttle, 1999). It has been calculated that the phosphorus concentration in the cell debris decreases by up to 87% due to the high phosphorus concentration of the phages. The amount of carbon and nitrogen in the cell debris corresponds to the concentration in the host cell (Jover et al., 2014). The released cell debris is re-used by the surrounding bacterial community (Middelboe, Jorgensen & Kroer, 1996). Thereby, the resource-supply shifts towards bacterial reproduction while energy fluxes to protists and higher trophic levels are hampered (Fuhrman, 1999).

Different phage strategies have been proposed, named “Killing-the-winner” (KtW), “Piggyback-the-winner” (PtW), and “Piggyback-the-loser” (PtL). Other phage strategies are “Piggyback-the-persistent”, describing a lysogenic phage strategy of constantly low host abundance (Paterson et al., 2019), and “Make-the-winner”, describing a prophage mediated viral defense (Dedrick et al., 2017; Knowles & Rohwer, 2017). In KtW, phages prevent the best bacterial competitor from building up high biomass and therefore ensure coexistence with other bacteria of lower competition strength (Thingstad & Lignell, 1997; Thingstad, 2000). Although the mechanism also applies for lysogenic phage infections (Thingstad & Lignell, 1997), the model used in previous studies describes a lytic phage infection (Thingstad, 2000). In PtW and PtL, temperate phages are characterized by a switch of lysogenic to lytic infection under different conditions. Under PtL, temperate phages switch from lytic to lysogenic infections if host abundance is low (Knowles et al., 2016), whereas temperate phages under PtW initiate lysogenic infections at high host abundances and growth rates (Silveira & Rohwer, 2016; Knowles et al., 2016). Several data sets have been published promoting either PtW (Silveira & Rohwer, 2016; Coutinho et al., 2017) or KtW (Liang et al., 2019) as the dominant phage strategy. However, different phage strategies could be favored depending on environmental conditions (Bongiorni et al., 2005; Weinbauer et al., 2009; Payet & Suttle, 2013). The density and metabolic activity of bacterial communities is affected by changes in the abiotic environment, which result in selective consequences for phages and their infection mechanism.

### Fluctuations in bacterial context

Most abiotic factors are not constant, but occur as random events or follow rhythms such as day-night cycles, tide systems, or seasonal weather conditions. In bacterial communities, these variations often imply effects of varying resource supply. Many ecosystems are affected by such fluctuations including seasonal ponds, intertidal ecosystems, groundwater systems, flood plains, and riparian forests. The frequency and strength of temporal variations have different effects, depending on the community they act on (Hutchinson, 1961). Fluctuations should have a great impact on bacterial communities, as their duration is longer than bacterial generation times (Gibson et al., 2018). However, microcosm-experiments have shown that resource fluctuations have a low impact on bacterial communities, but strong negative effects on their predators (Karakoç et al., 2017, 2018). We assume that different phage strategies show different advantages to overcome resource fluctuations. In our study, we analyze the impact of a fluctuating resource supply on different phage strategies.

The quest for understanding the driving forces of bacterial diversity is challenging because of the high complexity of natural food webs, displaying an interplay of various mechanisms. These processes are hard to observe and dissect in nature. Laboratory experiments can offer more controllability due to simplifications and the possibility of replicates. Simplified mathematical models allow to extract particular mechanisms and to investigate their patterns and their relevance to community dynamics. In our study, we simulate *in silico* the effect of different phage strategies on bacteria that are exposed to fluctuations in external resource supply by using ordinary differential equations (ODEs).

We ask how the three phage strategy promote bacterial coexistence under (a) stable, and (b) variable resource supply. The lytic and temperate phage strategies PtW and PtL were modeled by adapting the lysis rate of the infected bacteria. Additionally, we included a variable resource supply and the reuse of nutrients released by the viral shunt. With our model, we want to understand how environmental variability and different phage strategies shape bacterial population dynamics and coexistence.

Our results show that the viral shunt facilitates bacteria coexistence for all phage strategies by an enrichment of the system. Especially, the lytic infection and PtW allowed for bacterial coexistence at low resource supply, whereas PtL shifted the range of coexistence towards higher resource levels. The temperate phage strategy PtW yields bacterial coexistence in environments with low fluctuations over a limited range of resources, whereas PtL stabilizes population dynamics over a wide range of stable and fluctuating resource concentrations. We were able to show that the established lytic infection model provides resilience when faced with resource fluctuations. An increase of the bacterial growth rate leads to a stabilization of population dynamics at higher resource fluctuations for the lytic infection and the PtL model. As bacterial survival also benefits their phages, this result indicates that lytic phage infection, PtW, and PtL represent different phage survival strategies to cope with environmental variability.

## Methods & model

### General model assumptions

**Figure 1:**
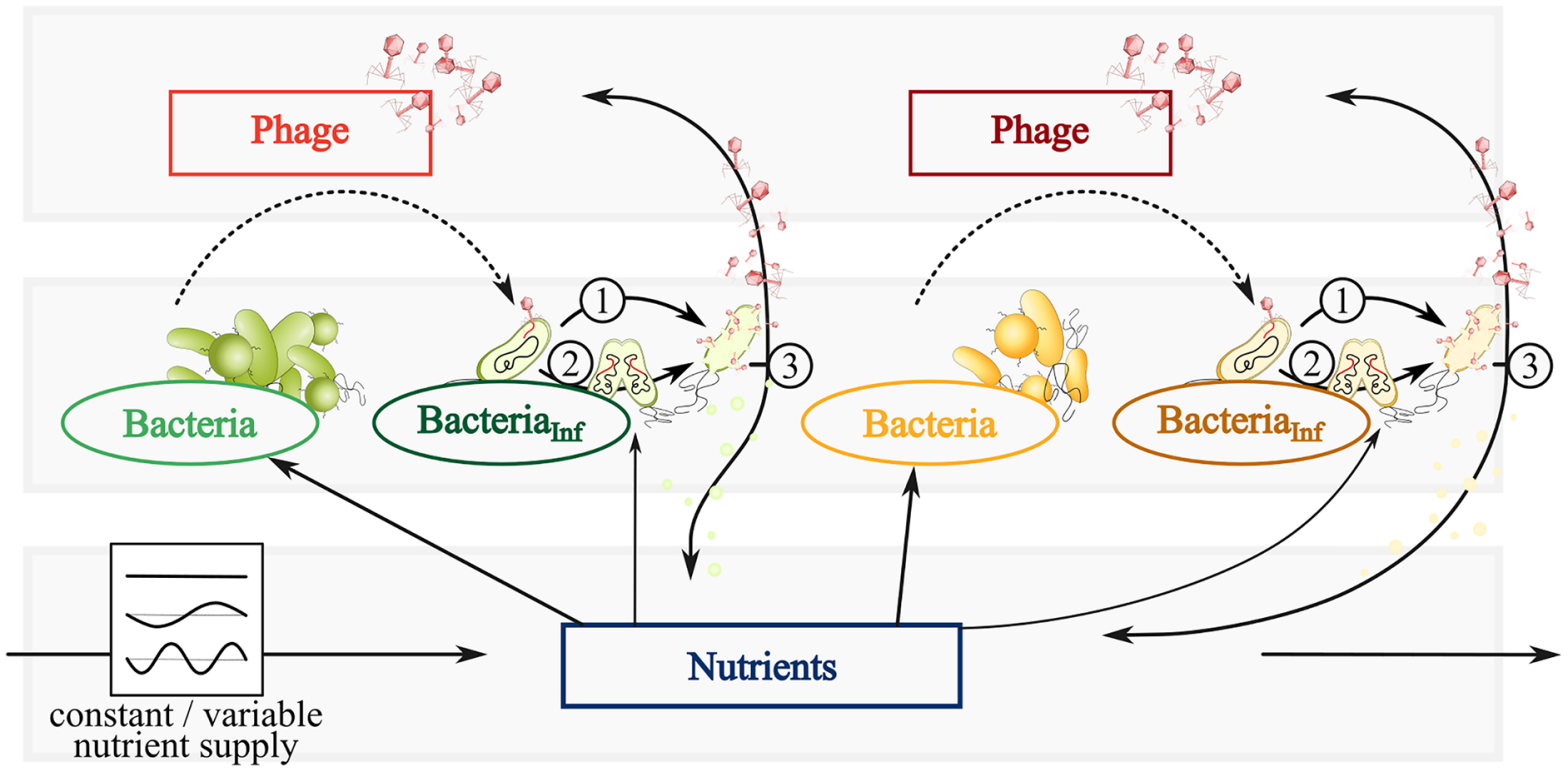
Conceptual graphic of a simplified bacteria-phage model with constant or fluctuating nutrient supply. Color code represents fast (green) and slow growing bacteria (yellow) feeding on the same nutrients (blue). Different phages (red or dark red) infect fast growing bacteria (dark green) and slow growing bacteria (brown). Dependent on the infection mechanism: (1) Phages inside infected bacteria enter the lytic cycle and lyse bacteria at a certain rate, thereby spreading phages and nutrients *via* the viral shunt. (2) Phage inside infected bacteria enter the lysogenic cycle and reproduce with its host as a prophage. The switch of lysogeny to lytic infection thereby variates with host abundances (PtW and PtL). (3) Lysis spreads phages and nutrients *via* the viral shunt, which can be consumed by bacteria.

The trophic interactions shown in Fig. 1 are quantitatively described by ordinary differential equations (ODEs). The time-dependent densities B_k_, I_k_, and P_k_ represent bacteria, infected bacteria, and phages of species k = 1,2, respectively. A nutrient density, N, extends the system to analyze the impact of fluctuating resources on the population dynamics.

### ODE—model

The bacterial growth in the absence of the phage was described by a Type 2 functional response (Monod, 1949) (Eq. (1)).

(1) $$G\left( N \right) = x{y_k}\displaystyle{N \over {N + {N_H}}}$$

The metabolic rate x for heterotrophic bacteria (Makarieva et al., 2008) was used to describe the bacterial growth rate scaled by a metabolic coefficient y_k_ and N_H_ is the half-saturation density. Both bacterial species k = 1,2 differ in their metabolic coefficient y_k_ to describe slow and fast growing bacteria (Table 2). The change of bacterial densities over time reads

(2) $$\dot{B}_k = G\left(N \right){B_k}-\underbrace{x\,B_k}_{metabolic\;loss} - \underbrace{i\,{B_k}\,{P_k}}_{loss\ via\,infection}.$$

Bacteria have a metabolic loss described by the metabolic rate x, which is assumed to be equal for both species k = 1,2, and i is the adsorption rate of phage P_k_.

**Figure 2:**
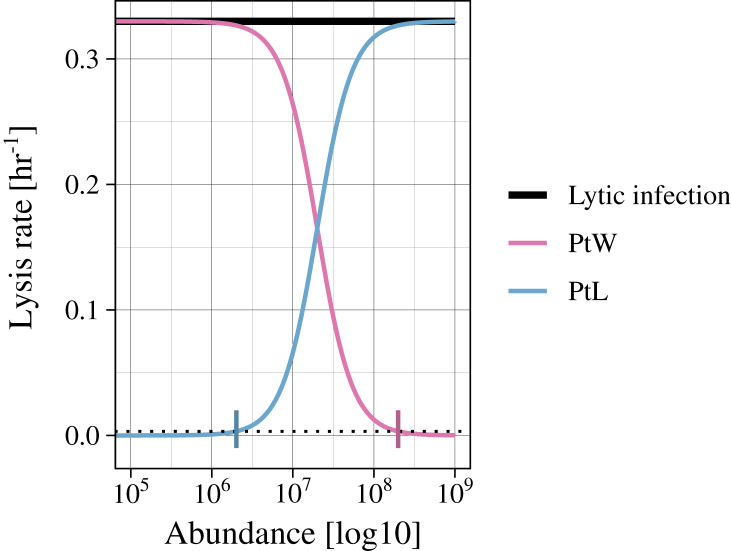
Variation of the lysis rate for different phage strategies. The lytic infection (black) has a constant lysis independent of host abundance. The temperate phage strategies switch between lysogenic and lytic infections and are therefore simulated with a density dependent phage induction rate. “Piggyback the winner” (pink) is defined by a lysogenic infection at high host abundances, the lysis rate is thereby suppressed. In contrast, “Piggyback the loser” (blue) is defined by a lysogenic infection at low host abundances. With increasing host abundance, the phages will enter the lytic cycle, so that the lysis rate will rise. A switching point was set to a lysis rate of s = 0.0033 [h^−1^] (black dotted line), turning the growth of infected bacteria on or off.

Infected bacteria are produced by bacteria getting infected by a phage. In the case of a lytic infection, the infected bacteria have no own reproduction anymore. For the lysogenic infection of a bacteria, growth is enabled (as in Eq. (1)). We include a switch between the lysogenic and lytic life cycle for both temperate phage strategies (PtW and PtL), enabling growth or not, respectively. The switching point is set to a lysis rate of 0.0033 [h^−1^], which is reached at an abundance of non^-^infected and infected bacteria of 2.01*10^6^ for PtL and 1.99*10^8^ for PtW (Fig. 2; Figs. S1, S2).

(3) $$\dot {I}_k = \underbrace{({1 + c})i{B_k}{P_k}}_{gain\;of\;infection} + \underbrace{G( N ){I_k}}_{growth\ lysogenic\ bacteria} - \underbrace{x{I_k}}_{metabolic\;loss} - \underbrace{s{I_k}}_{loss\ via\;lysis}$$

The lysis of infected bacteria releases a defined fraction of phage biovolume, n, and nutrients, (1 − n), that can be reused by the bacteria (*i.e*. the viral shunt). This lysis was defined by the lysis rate, s.

Phage infection happens by chance and is described as a linear function with the adsorption rate, i. We chose the same adsorption rate for both phages infecting slow and fast growing bacteria. The infection of bacteria was defined as in Beretta & Kuang (1998); Gulbudak & Weitz (2019). It is assumed that, although many phages attach to a bacterial cell, only one phage is responsible for the infection. We used a conversion factor, c, to resize the volume of bacteria to phages for infection (Eq. (3), (4)), because we used the biovolume [µm^3^ mL^−1^] for phage and bacteria (Table 1). Phages can become inactive by different chemicals or physical environmental factors, such as unfavorable temperatures, pH, salinity, or ions (Jończyk et al., 2011). That is described by the decay rate, d. Combined, these processes define the rate of change for phage densities over time.

**Table 1:** Normalized densities used for simulations.

Initial densities			norm. Values	References/Comments
Bacteria (B_k_)	8.30 * 10^6^	particles mL^−1^	8.30 * 10^−1^	(Beretta & Kuang, 1998)
Infected bacteria (B_k_)	1.70 * 10^6^	particles mL^−1^	1.70 * 10^−1^	(Beretta & Kuang, 1998)
Phages (P_k_)	1.00 * 10^7^	particles mL^−1^	3.10 * 10^−4^	(Beretta & Kuang, 1998)
Nutrient (N)			0.60–30.00	
Supply concentration (N_0_)			0.60–30.00	
Half-saturation density (N_H_)	1.00 * 10^7^	particles mL^−1^	1.00	
Switch point PtW	1.99 * 10^8^	particles mL^−1^	19.8997500	
Switch point PtL	2.01 * 10^6^	particles mL^−1^	0.2010076	
Half-saturation density (SH)	4.00 * 10^7^	particles mL^−1^	4.00

(4) $$\dot {P}_k = \underbrace{ns{I_k}}_{phage\;release} - \underbrace{ci{B_k}{P_k}}_{loss\;via\;infection} - \underbrace{d{P_k}}_{phage\;decay}$$

Resource change over time was simulated by using a chemostat model with a turnover rate, D, and inflow supply concentration, N_0_,

(5) $$\dot N = D( {{N_0} - N}) - \underbrace{G(N){B_k}}_{bacterial\;consumption} - \underbrace{G(N){I_k}}_{lysogenic\;bacterial\;consumption} + \underbrace{( {1 - n})s{I_k}}_{viral\;shunt}$$

The consumption of resources by bacteria is described by the Type 2 functional response as in Eq. (1). The reuse of nutrients caused by the viral shunt is included subtracting the fraction of released phage biovolume, n, (Weitz, 2015) of the total amount of the lysate, s I_k_. The nutrients released by the viral shunt are thereby included to the total nutrient supply and can be consumed by all growing bacteria.

### External fluctuations

To include an external fluctuation of resources, a sine curve was used to modulate the turnover rate, D, over time t. The sine curve is defined by the amplitude, a, and the period, T, describing the fluctuation around the mean resource supply, D. We used a < 1 to guarantee positivity.

(6) $${D_F} = \left( {aD\ sin\left( {\displaystyle{{2\pi } \over T}t} \right) + D} \right)$$

### Defining phage life cycles by changing the lysis rate

The phage induction through different phage strategies is simulated by altering the lysis function. The lytic infection is described by a constant lysis rate, s, as in previous studies (Beretta & Kuang, 1998; Gulbudak & Weitz, 2019). The temperate phages are characterized by a change of lysogenic to lytic infection. The PtL phage strategy describes lysogenic infections at low host abundances (Knowles et al., 2016). At increasing host abundances the phage enter the lytic cycle, which we described by an increasing lysis rate (Eq. (7)). The PtW phage strategy argues the converse (Knowles et al., 2016), which we specified as a suppressed lysis rate by more lysogenic infections at high host abundances (Eq. (8)). We transformed the constant lysis rate, s, into a rate dependent on the host density to simulate phage induction for both temperate phages. We use an increasing and a decreasing sigmoidal function for PtL and PtW, respectively (Fig. 2). For comparability, we fixed s = 0.330 [h^−1^] as the maximum lysis rate for all phage strategies (Fig. 2; Figs. S3, S4). The half-saturation density, SH, for PtL was fixed to SH = 4*10^7^ (Fig. S3). The parameters for PtW were set to r = 0.5 and H = 1 (Fig. S4).

(7) $${s_{PtL}} = \displaystyle{{s{{\left( {{B_k} + {I_k}} \right)}^2}} \over {{{\left( {{B_k} + {I_k}} \right)}^2} + SH}}$$

(8) $${s_{PtW}} = \displaystyle{s \over {{{\left( {\left( {{B_k} + {I_k}} \right)r} \right)}^2} + H}}$$

### Parameter settings

The model’s initial densities of bacteria and phages, parameters and conversion rates used for simulations are shown in Tables 1 and 2. For our initial values we choose the ratio of 0.17 to 0.83 for infected to non-infected bacteria (Beretta & Kuang, 1998). Other quantities such as the inflow resource concentration (N), supply rate (N_0_), and half-saturation density (N_H_) were set to a parameter range, that created stable population dynamics. We transformed the densities of bacteria and phages into biovolume (Table S1), and normalized them by the half-saturation density (N_H_). Different initial states were tested and did not affect the results of the simulations. Therefore, the same initial states were used for all calculations (Table 1). After numerical simulation of the model, the normalized biovolumes of bacteria and phages were counted back into abundance for a further evaluations.

### Rates

To describe the growth and metabolic loss of the bacteria we used a metabolic rate for heterotrophic bacteria (Makarieva et al., 2008), which we set to 40 [J (s * kg)^−1^]. We transformed this rate to equalize the units [h^−1^] using the conversion rates of (Peters, 1983). The virus decay rate was fixed to d = 0.0866 [h^−1^] and the lysis rate to s = 0.330 [h^−1^] (Gulbudak & Weitz, 2019). The phage adsorption rate was set to i = 1.64*10^−10^ [mL h^−1^], for both phages infecting slow and fast growing bacteria. This rate had to be normalized, because we used the normalized biovolume of bacteria and phages for calculations (Table S1). All rates used for simulations are listed in Table 2.

**Table 2:** Rates [h^−1^] and conversion rates used for all model simulations.

	Values	Units	norm. Values		References/Comments
**Rates**					
Turnover rate (D)	1/24	h^−1^			resource turnover/mean level for fluctuations
Metabolic rate (x)	0.0206	h^−1^			adapted after (Makarieva et al., 2008)
Lysis rate (s)	0.3300	h^−1^			(Gulbudak & Weitz, 2019)
Phage decay (d)	0.0866	h^−1^			(Gulbudak & Weitz, 2019)
Adsorption rate (i)	1.64 * 10^−10^	mL h^−1^	5.23	h^−1^	
**Conversion rates**					
Metabolic scaling constant (y_k_)	y_1_ = 3.75/7.5/15; y_2_ = 2/4/8				
Phage burst volume (n)	0.02				(Weitz, 2015)
Conversion factor viral infection (c)	3.14 * 10^−4^				Ratio of bacterial-virus biomass
PtW: correction parameter (r)	0.50				
PtW: correction parameter (H)	1.00				
Amplitude (a)	0.00–0.99				
Period of oscillation (T)	24/162/720/8,760	h			

### Conversion rates

Constants scale different parameters, such as the metabolic scaling constant, y_k_, to specify bacteria growth. We fixed y_1_ = 7.5 and y_2_ = 4 to define fast and slow growing bacteria, respectively (Brose, Williams & Martinez, 2006). All other parameters are identical for both bacteria species and their associated phages. The ratio of phage to bacteria biovolume, c, was used to adjust the loss of phage biovolume during the adsorption by the host. The phage burst volume, n, has been set to 0.02 (Weitz, 2015). All values of the conversion rates used for simulations are listed in Table 2.

The model was simulated using R 3.6.1 (R Core Team, 2020) on Ubuntu 18.04.5 LTS. The packages *odeintr_1.7.1* (Keitt, 2017) and *EMD_1.5.8* (Donghoh & Hee-Seok, 2009, 2018) were used for simulations. We used the solver “rk54_a”. For the graphical output the packages *ggplot2_3.3.0* (Wickham, 2016), *viridis_0.5.1* (Garnier, 2018), *imager_0.42.3* (Barthelme, 2020), and *gridExtra_2.3* (Auguie, 2017) were used. The model code is available at the github repository https://github.com/Es-Vo-26/Phage-Infection-Strategies.

## Results

### Phage strategies

**Figure 3:**
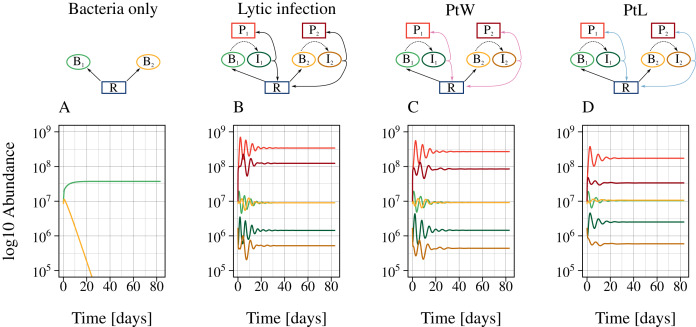
The presence of phages enables bacterial coexistence. Bacterial coexistence without and with different phage infection mechanisms was tested. The graphic shows time series [days] of the different phage-bacteria models with a constant resource supply, N_0_ = 2. Equal parameters and initial values were chosen for all simulations (Table 1), with a metabolic scaling constant of y_1_ = 7.5 for fast growing, and y_2_ = 4 for slow growing bacteria. Color code according to Fig. 1.

**Figure 4:**
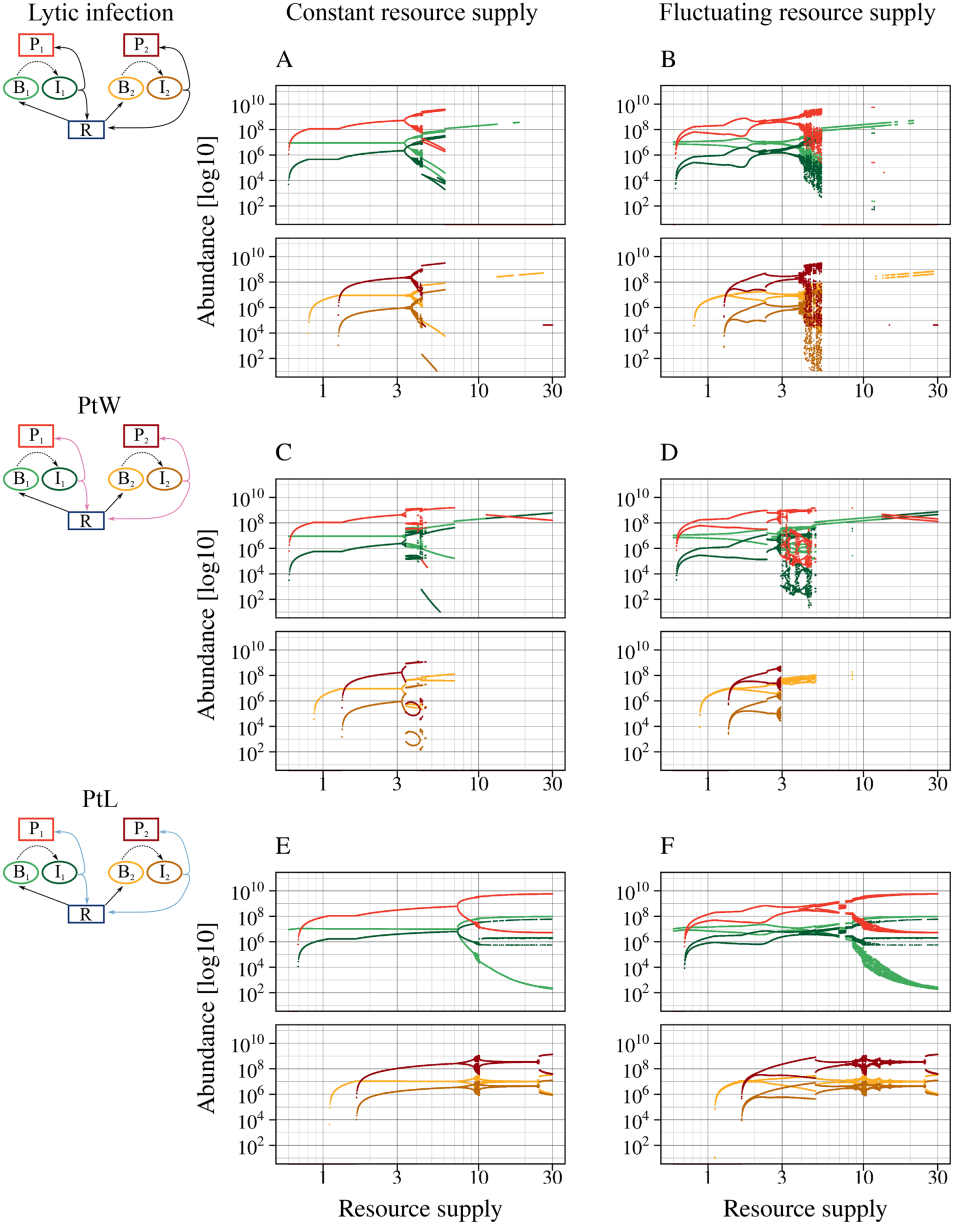
All phage strategies promote bacterial coexistence over different resource supply ranges. A fluctuating resource supply reduces the range of coexistence. Bifurcation diagrams of the different phage-bacteria models are shown with a constant and fluctuating resource supply varying between 0.6 and 30. Equal parameters and initial values were chosen for all simulations (Table 1), with a metabolic scaling constant of y_1_ = 7.5 for fast growing, and y_2_ = 4 for slow growing bacteria. An amplitude of a = 0.5 and a period of T = 7 days was chosen to simulate fluctuating resources. Upper graphs show fast growing bacteria, bottom graphs the slow growing bacteria and the associated phage infection. Color code according to Fig. 1.

The different phage strategies were modeled by changing a constant lysis rate to a density-dependent phage inductions rate (Fig. 2). Additionally, bacterial reproduction was enabled for lysogenic but turned of for lytic phage strategies by including a switching point (PtW, PtL). First, we calculated different time series with a fixed set of parameters (Tables 1, 2) without and with phage infecting bacteria. Without infection, the bacteria with a slower growth rate were outcompeted by the faster-growing bacteria (Fig. 3A). Coexistence was enabled in the presence of the corresponding phage for all phage strategies (Figs. 3B, 3C, 3D). Next, we used bifurcation diagrams to analyze the effect of the viral shunt (Fig. S5) and to compare the stability of the different phage strategies (Fig. 4). For evaluation of the system stability we examined coexistence of slow and fast growing bacteria, persistence of the phage infection, oscillations, and species extinction over a resource supply, N_0_, systematically varying between 0.6 and 30. By including the viral shunt (Eq. (5)) into the model the system got enriched. Thereby, slow and fast growing bacteria could coexist at lower resource concentrations, and population oscillations at high resource levels have been shifted to lower resource levels (Fig. S5). The viral shunt was included to all phage strategies.

The lytic and the temperate phage model PtW resulted in coexistence of slow and fast growing bacteria at lower resource supply (Figs. 4A, 4C). In comparison, bacterial coexistence was shifted to a higher resource level for the temperate phage model PtL (Fig. 4E). At higher resource supply, populations starts to oscillate, which is known as the paradox of enrichment (Rosenzweig, 1971).

The different phage strategies featured considerable variability in coexistence and stability. The proportion of infected bacteria was increased for PtL (Fig. 4E) compared to the purly lytic infection and PtW (Figs. 4A, 4C). That results from the lysogenic infection at low host densities allowing infected bacteria to reproduce and reaching high abundances if the resource supply increases. The temperate PtW and the lytic strategy showed oscillating population dynamics already at medium resource supply (Figs. 4A, 4C). For the temperate model PtW the fast growing non-infected bacteria went extinct at high resource concentrations over N_0_ = 11.23, whereas the associated infected bacteria and the phage persisted (Fig. 4C). The PtL model displayed large oscillations of the fast growing bacteria at high resource supply. In contrast, the slow growing bacteria with the infection showed weaker oscillations (Fig. 4E).

We repeated the analysis using an oscillating turnover rate. The relative amplitude a = 50% and the period T = 7 days were kept fixed and nutrient supply N_0_ was systematically varied from 0.6 to 30 (Figs. 4B, 4D, 4F). The point of bacterial coexistence and persistence of the phage infection for low resources was shifted to higher resource levels, whereas overall species extinction for high resources was shifted to a lower resource level. Thus, resource fluctuations reduced the range of bacterial coexistence for all phage models (Figs. 4B, 4D, 4F). Similar to a constant resource supply the temperate phage model PtW resulted in an extinction of the fast growing non-infected bacteria at high resource levels, whereas infected bacteria were able to persist (Fig. 4D).

### Low periods and amplitudes do not affect bacterial systems

**Figure 5:**
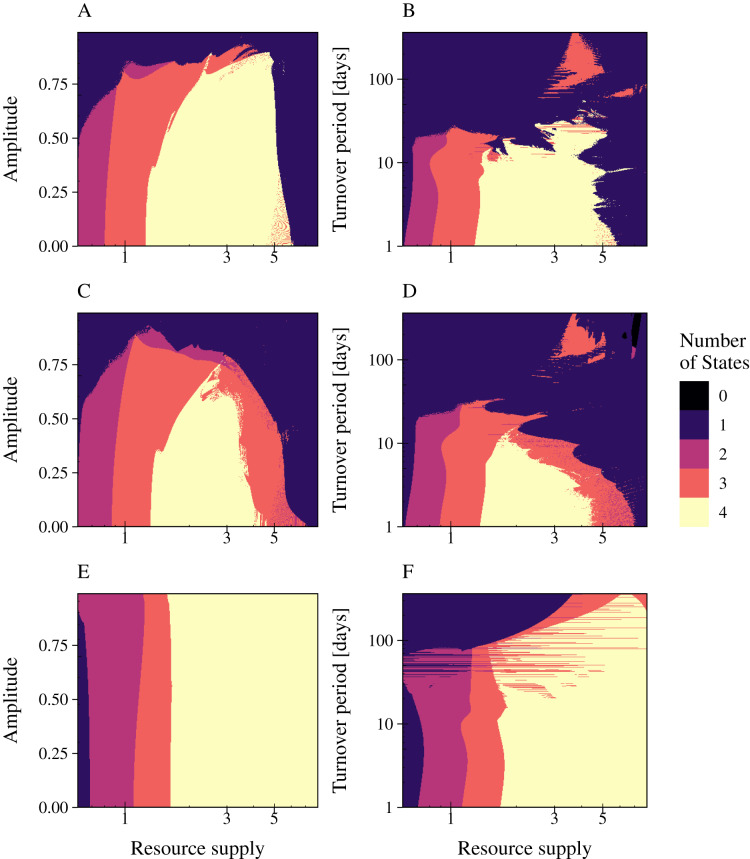
Resource fluctuations reduce the range of bacterial coexistence and the persistence of the infection at low resource concentrations and high turnover periods. The number of persisting states is shown for varying rates of resource amplitude or period over resource supply for the lytic and the temperate phage strategies PtW and PtL (rows). A color code indicates the number of persisting states: (0) no species coexist (black); (1) fast growing bacteria (blue); (2) fast growing bacteria and their infection (purple); (3) fast and slow growing bacteria and the infection of the fast growing bacteria (red); and (4) fast and slow growing bacteria and their infections persist (yellow). Phage and infected bacteria were summarized as one common state. Column 1: Amplitude is given as the percentage of the mean resource supply of 1/24 h^−1^. The period was set to 30 days of one resource turnover. Column 2: The period is stated in turnover days, increasing on a logarithmic scale. The amplitude was set to 0.9.

We investigated the impact of resource fluctuations on population stability by simulating the three types of phage strategies while (a) independently varying relative amplitude a (0% to 99%) and nutrient supply N_0_ (period fixed at T = 30 days); and (b) independently varying period T (1 day to 1 year) and nutrient supply N_0_ (relative amplitude fixed at a = 90%) (Fig. 5; Figs. S6, S7, S8). The nutrient supply N_0_ was varied from 0.6 to 8. The number of persisting states was plotted in a 2D graphic for (a) increasing values of amplitude or (b) period plotted over an varying resource supply. Since phages always occur with their associated infected bacteria in our model results, we summarized phage and infected bacteria as one common state. The color depicts biodiversity, representing the different model states from black (all bacteria and infections extinct) to yellow (all bacteria and their infections can persist). Our results showed that all phage strategies were not affected in their range of coexistence by an amplitude of 50% (Fig. 5 Column 1). Similar, a resource supply period of T = 7 days did not change the range of coexistence for all tested phage models. The combination of high amplitude with a reduced resource supply period and low resource concentrations led to a collapse of bacterial populations. Here, phage infection of the slow growing bacteria went extinct first (red area), followed by slow growing bacteria (purple area) and the phage infection of the fast growing bacteria (blue area) (Fig. 5). Bacterial coexistence and phage persistence (yellow area) was not possible at low resource concentrations.

The phage models showed a great variance in their coping with resource fluctuations (Fig. 5). In comparison with PtL, the lytic infection and the temperate infection PtW enabled bacterial coexistence and persistence of the infection over a limited range of resource supply. Here, the temperate phage mechanism PtW was most sensitive to resource fluctuations (Figs. 5C, 5D). Especially low resource concentrations and an increase of the turnover period over 7 days for PtW or over 10 days for the lytic infection limited bacterial coexistence (Figs. 5B, 5D). For the temperate phage model PtL bacterial coexistence and persistence of the phage infection were also reduced at low resource concentrations (Figs. 5E, 5F). At a certain range of the nutrient turnover period (T = 30 to T = 70 days) the phage infection of the fast growing bacteria got stabilized due to the resource fluctuations (Fig. 5F). In contrast to the lytic infection or PtW, the PtL model showed a stable bacterial coexistence and persistence of the phage infection over a broad range of resource supply and fluctuations (Figs. 5E, 5F). Thus, PtL could be a preferred phage strategy in environments with fluctuating resource conditions.

### Interaction strength enables coexistence at higher resource fluctuations

**Figure 6:**
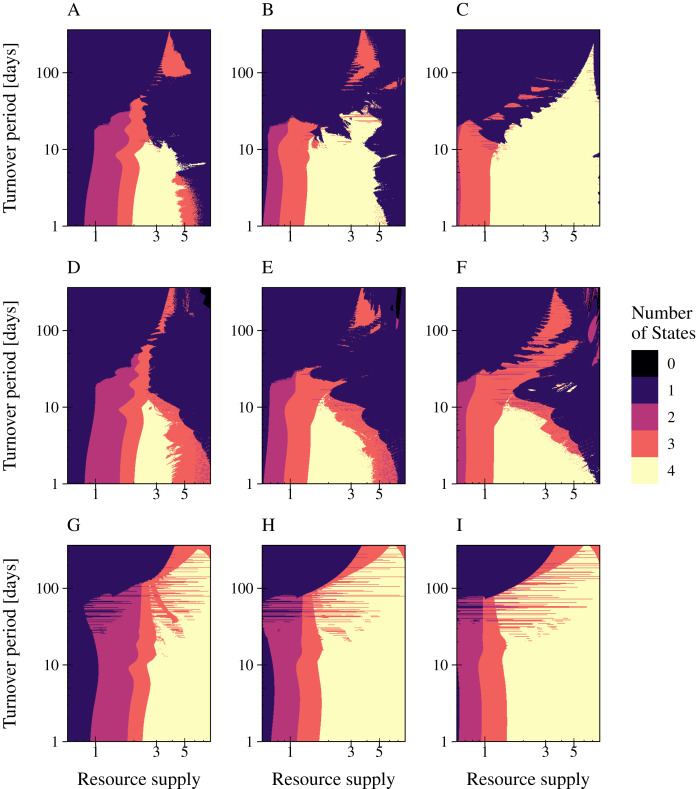
High bacterial growth rates increase the resilience against nutrient variability for the lytic infection and the phage strategy PtL. The number of persisting states is shown at a varying resource period over resource supply for the lytic and the temperate phage strategies PtW and PtL (rows). The period is stated in turnover days, increasing on a logarithmic scale. The amplitude was set to 0.9. Low growth rates (left) were simulated with a decreased metabolic scaling constant (y_1_ = 3.75; y_2_ = 2), higher growth rates (right) by an increased metabolic scaling constant (y_1_ = 15.0; y_2_ = 8.0). For the normal growth (center) metabolic scaling constants of y_1_ = 7.5 and y_2_ = 4 were used. A color code indicates the number of persisting states: (0) no species coexist (black); (1) fast growing bacteria (blue); (2) fast growing bacteria and their infection (purple); (3) fast and slow growing bacteria and the infection of the fast growing bacteria (red); and (4) fast and slow growing bacteria and their infections persist (yellow). Phage and infected bacteria were summarized as one common state.

A change in interaction strength such as a more efficient infection by phages or uptake of resources by bacteria had an impact on species coexistence (Fig. 6). We investigated the impact of interaction strength on population stability by simulating the three types of phage strategies. Low interaction strength was simulated by a decrease of bacteria growth rates (y_1_ = 3.75; y_2_ = 2), whereas a more efficient interaction was described by increased growth rates (y_1_ = 15.0; y_2_ = 8.0). The values of Table 1 were used to define the medium interaction strength (as in Fig. 5). The number of persisting states was plotted in a 2D graphic by varying the fluctuation period (1 day to 1 year) over an increased nutrient supply N_0_, varying between 0.6 and 8 (relative amplitude fixed at a = 90%) (Fig. 6). The change of bacterial growth rates altered the resource supply range of species coexistence. General low growth rates reduced the range of bacteria coexistence and the persistence of the phage infection (Figs. 6A, 6D, 6G). whereas general high growth rates expand the range of coexistence (Figs. 6C, 6F, 6I). Furthermore, a high growth rate enabled bacteria coexistence at higher resource fluctuations for the lytic and the PtL model, whereas low growth rates led to a decline of the biodiversity when resource fluctuations increase. Only for the temperate phage model PtW an increase in bacterial grow did not affect bacterial coexistence at higher resource fluctuations.

## Discussion

We performed *in silico* experiments with three different bacteria-phage models, to investigate the ability of different phage strategies to facilitate the coexistence of bacteria competing for limiting resources. In their natural environments, microbial communities are exposed to temporal variations in habitat conditions such as seasonality, rainfall, or tidal rhythms. However, fluctuations in resource supply should have disruptive effects on population dynamics, because bacteria are strongly coupled to their environment (Song et al., 2016; Muscarella et al., 2019).

### The viral shunt facilitates bacterial coexistence at low nutrient levels

As a first step, the viral shunt, as a result of bacterial lysis by phages, was included in all our bacteria-phage models. The fraction of phages in the viral shunt make up 0.01% to 0.02% of the total bacterial biomass (Weitz, 2015) and therefore change the stoichiometry of the cellular debris (Jover et al., 2014). The high phosphorus content of phages results in a decrease of phosphorus in the viral shunt, if more phages are released. The carbon and nitrogen content of the viral shunt is proposed to correspond to that of the lysed host (Jover et al., 2014). Studies show that almost 30% of the phytoplankton carbon is released by viruses (Talmy et al., 2019). The released nutrients are consumed by bacteria and increase their reproduction in lytic bacteria phage systems (Fuhrman, 1999; Shelford et al., 2012). An increase in available nutrients thereby enables bacteria to change their environment faster, leading to stronger population-level effects on their resources and consumers. In extreme cases, this leads to the extinction of community members and biodiversity loss (Ratzke, Barrere & Gore, 2020). In our study, we demonstrate that the same mechanism acts in the temperate phage strategies PtW and PtL.

Lysogeny is proposed as a survival strategy for phages in oligotrophic environments (Stewart & Levin, 1984), but also occurs frequently at high host densities (Silveira, Luque & Rohwer, 2021). Our results show, that the lytic infection and the temperate infection PtW enabled bacterial coexistence and stable infection dynamics at low nutrient concentrations. The PtW model is defined by lytic infection at low host abundances, which results in increased nutrient availability due to the viral shunt. This enrichment due to a lytic phage infection shifts also the persistence of infected bacteria to a lower nutrient level. In contrast, PtL shows lysogenic infection, if host abundance is low. The lysogenic bacteria can reproduce and thereby increase the competition for limited resources, which shifts the range of bacterial coexistence to higher resource levels. In laboratory experiments it was shown that the lysis of lysogenic infected bacteria is stochastic to some extent (Dennehy & Wang, 2011). For PtL, showing an increased fraction of lysogenic bacteria, this effect can also lead to a slight enrichment. We propose that lytic phage strategies promote bacterial coexistence, stabilize phage infections and thereby lead to increased diversity in oligotrophic systems.

Studies have shown a collapse of cyanobacteria communities in lakes or marine environments in response to nutrient enrichment. These collapses are related to an increased abundance of cyanophages (Gons et al., 2002; Coello-Camba et al., 2020). Our results show that phage infections destabilize bacterial communities by the viral shunt at eutrophic conditions. Through enrichment by the viral shunt, population dynamics oscillate already at lower nutrient concentrations, which is known as the paradox of enrichment (Rosenzweig, 1971).

### Temperate phage strategies show different patterns in population dynamics

We focused on modeling three phage strategies and analyzed them regarding their stability at increased energy input and resource fluctuations. The lytic infection (Beretta & Kuang, 1998; Gulbudak & Weitz, 2019) and temperate phage strategies PtW and PtL (Knowles et al., 2016) were modeled by a change of the lysis rate. For the temperate phage strategies we modeled a density dependent phage induction rate. Furthermore, we included a switch point to allow lysogenic infected bacteria to grow and reproduce with a prophage inside their genome, but turn off the growth for lytic infected bacteria.

The temperate phage mechanism PtL switches from a lysogenic to a lytic phage infection if host density increases. Our results show that PtL yields more stable populations and facilitates species coexistence across a broad range of resource supplies from oligotrophic to eutrophic conditions. Under PtL the abundance of infected bacteria is increased compared to the lytic model or PtW.

The lysogenic infection at low host abundances causes this effect, allowing more infected bacteria to replicate with their prophage. At higher host abundances more phages are produced due to an increase of the lysis rate, which controls bacterial density and competition. The PtL mechanism acts as a top down control for bacteria and stabilizes population dynamics. This results in stable coexistence of slow and fast growing bacteria, where fast growing bacteria show stronger oscillations due to a stronger top down control by their phages. Interestingly, studies have shown that better growth conditions enabled by high resource availability can lead to beneficial mutations in the bacterial genome (Wahl & Zhu, 2015), resulting in bacterial resistance against phage infection (Gomez et al., 2015). Our model suggests that the PtL mechanism can create an evolutionary pressure on bacteria under eutrophic conditions, potentially leading to bacteria resistance against phages.

The temperate phage mechanism PtW is characterized by an increased lysogenic phage infection at high host abundances (Knowles et al., 2016). Our results show that PtW leads to a destabilization of population dynamics. As expected, the fraction of infected bacteria increases at high resource concentrations, since more available nutrients lead to a higher reproduction of the bacterial host. During a lysogenic infection, the host replicates with its prophage, but is not directly killed by the phage. The phage cannot act as a top-down control on bacteria anymore. This increases the resource competition between non-infected and lysogenic infected bacteria as an indirect effect and thereby destabilizes population dynamics. At high resource availability PtW leads to the extinction of the fast growing non-infected bacteria, whereas the infected bacteria and phage persists. These infected bacteria are lysogenic infected bacteria, because their abundance is above the included switch point. In our model the lysogenic infected bacteria can outcompete non-infected bacteria because of the high phage number infecting bacteria at high resource levels. In natural systems the number of phage coinfections increases at high microbial abundances, which favors phage integration and thus lysogenic infections (Luque & Silveira, 2020; Silveira, Luque & Rohwer, 2021). A phage integration into bacterial genome at high host densities can have advantages for bacteria. It has been shown that lysogeny can alter the host’s genome and cellular processes (Menouni et al., 2015; Feiner et al., 2015; Howard-Varona et al., 2017). Thereby, phages can slow down diversification and increase genetic similarity in the host population (Weinbauer & Rassoulzadegan, 2004). In a stable environment, this can increase the survival chances of bacteria and its related phages. Alternatively, integration of phages due to lysogeny can also promote adaptation and increase diversity (Weinbauer & Rassoulzadegan, 2004), which can help the bacterial host to acquire new ecological niches.

### High interaction strength helps to overcome resource fluctuations

We also compared the stability of the different phage strategies under fluctuations by modifying the resource inflow into the system. For this purpose, the amplitude and turnover period of the mean resource supply were independently varied.

In natural soil systems, bacteria depend on an inflow of water and resources. It has been shown that bacterial species richness is highest at intermediate water contents (Bickel & Or, 2020). High rainfall events lead to highly connected systems, where competition in the community is strong. This causes more negative microbial interactions, leading to a loss of biodiversity (Ratzke, Barrere & Gore, 2020). Our results show that amplitudes up to 50% of the mean resource supply do not affect biodiversity. Higher amplitudes in combination with a decreased turnover period of over 7 days lead to a decline in bacterial coexistence and stable infection dynamics. Here, phages are more affected by resource fluctuations. This is in line with microcosm-experiments showing that fluctuations in resource availability have a more negative impact on predators than on their prey (Karakoç et al., 2018). Our results show that species can persist at higher resource fluctuations if their growth rate is increased. High resource fluctuations at a low mean resource supply only allow fast growing bacteria to persist in our model, independent of the phage strategy. A stronger interaction to their environment due to faster growth rates or more efficient use of resources can help bacteria to react to variable environments. A fast adaption and effective interaction within variable and oligotrophic environments are essential for bacterial survival.

### Seasonality of phage strategies

Many studies demonstrate that infections show patterns of seasonality, that can be caused by a change in environmental variation (Fisman, 2012; Hwang et al., 2017; Girard et al., 2020). The three phage strategies analyzed in our study are assumed to be favored at different resource levels or different time points in the year (Payet & Suttle, 2013). Other studies (Maurice et al., 2013) demonstrated less evidence for seasonality of lysogenic interactions but a strong dependence of lytic infections on resource concentrations. All phage strategies tested in our model feature destabilization at high resource fluctuations at a low mean resource supply. Other mechanisms can help to persist under variable resource conditions and contribute to the stability of bacterial communities, such as bacteria dormancy (Jones & Lennon, 2010). Our results show that faster exploitation of resources enabled bacteria existence and persistence of their infection despite high fluctuations. A lytic phage infection at low host densities can enrich oligotrophic systems by the viral shunt and thereby promote bacterial diversity. This effect is enhances for high bacterial growth rates. However, only PtL showed persistence at high resource fluctuations and therefore can act as a main phage strategy in highly variable environments.

Increases in resource concentrations and fluctuations lead to diverse patterns in lytic and temperate phage strategies (PtW, PtL). All models enable bacterial coexistence over a broad range of resource supply and substantial variance in fluctuations. The temperate phage strategy PtW and the lytic infection are more dependent on resource supply than the temperate PtL model. Interestingly, the purely lytic phage infection enabled bacterial coexistence and phage persistence over a broad range of nutrient fluctuations. This effect was actually strengthened by a increase in bacterial growth rate for high levels of nutrient supply.

The lytic phage infection depends on host metabolism and increase with host activity (Maurice et al., 2013). Our results show, that an increase in host growth rate not only supports the lytic phage strategy but also the resilience against nutrient variability. That makes the lytic infection more sensitive to environmental conditions and seasonality.

The temperate phage model PtW is particularly vulnerable to enhanced resource concentrations and fluctuations, which lead to a decline in biodiversity. Our results suggest that PtW should be favored at low resource conditions with moderate environmental variability. This could be met in highly diverse environments, where other predators such as protists act as a top-down control for bacteria abundance. PtW could benefit their highly abundant hosts by promoting their genetic adaptation due to the lysogenic infection (Weinbauer & Rassoulzadegan, 2004). Thereby, the PtW phage strategy would not necessarily depend on seasonality.

In the last decades, anthropogenic influences caused climatic changes, affecting seasonal patterns such as temperature, and the amount and days of rainfall. Furthermore, variable and extreme weather events are proposed to increase in the following decades (Intergovernmental Panel on Climate Change, 2014). The main drivers for microbial community composition are the indirect effects of climate change such as soil water content or resource availability (Deltedesco et al., 2020). Bacterial communities are coupled to their environments (Song et al., 2016; Muscarella et al., 2019), which makes the community structure highly sensitive to anthropogenic impacts (Rocca et al., 2019). Our simulations show that a reduced resource availability due to prolonged turnover periods leads to a rapid decline in bacterial coexistence. This effect would be increased in structured environments, such as soil, due to limited diffusion of phage particles (Sousa & Rocha, 2019). Therefore we argue that increasing dry periods caused by climate change would have disastrous effects on bacteria communities. Especially oligotrophic environments would experience a decline in biodiversity. However, these aspects are potential hypotheses for future studies.

## Conclusion

Various mechanisms drive bacterial diversity, and it remains an ongoing challenge to disentangle and understand their relative importance for bacterial coexistence. Model systems can facilitate such understanding by segmenting complex interactions into simplified mathematical models. We analyzed how different phage strategies shape bacterial coexistence and persistence across gradients in resource supply and fluctuations. We developed a microbial trophic network model for lytic and temperate (PtW, PtL) phage strategies and performed ‘*in silico*’ experiments.

The lytic infections and PtW enabled bacteria coexistence at low resource concentrations by an enrichment of the system *via* the lysis-driven release of nutrients. Bacteria are highly coupled to their environment and are thereby affected by long periods of resource turnover, especially if resource concentration is low. Surprisingly, the purely lytic infection yields stable bacterial coexistence despite strong resource variability. An increase in bacterial growth amplified this effect, suggesting that host activity not only supports the lytic phage infection but resilience to environmental variability. The temperate phage strategy PtW leads to a destabilization of population dynamics at high resource concentrations or fluctuations. This effect is caused by a stagnated lysis at high host densities, generating a reduction in top-down control of the bacterial community. However, increased integration of phages into the host genome can lead to a better adaption to new environmental conditions (Weinbauer & Rassoulzadegan, 2004). In contrast, the temperate phage strategy PtL enabled bacteria coexistence over a wide range of resource concentrations and fluctuations. PtL could occur as the main phage strategy in environments with large variations in resource availability, such as aquifers or deep soil habitats. Finally, our results point out that all phage strategies define independent strategies to overcome environmental variability. Our study highlights the importance of bacteria-phage interactions for the maintenance of microbial diversity and ecosystem functioning under high environmental variability.

## Supplemental Information

10.7717/peerj.12194/supp-1 Supplemental Information 1 Supplemental methods and results.

10.7717/peerj.12194/supp-2 Supplemental Information 2 Initial model abundances and densities converted into biovolume and normalized by the half-saturation density N_H_.

10.7717/peerj.12194/supp-3 Supplemental Information 3 The switching point selected for PtL does not affect bacterial coexistence or phage infection persistence. Sensitivity analyses for the switching point a) at a lysis rate of 0.001 [h^−1^] b) at a lysis rate of 0.0033 [h^−1^] c) at a lysis rate of 0.165 [h^−1^]. The number of coexisting species is shown for increased values of the switching point over a constant and fluctuating resource supply (T = 30 days; a = 0.9). The number of persisting states is given with a color gradient from no state persist (black) to all states – slow and fast growing bacteria, as well as their associated phage and infected bacteria—can persist (yellow). Bifurcation diagrams show the population dynamics for the three switching points.

10.7717/peerj.12194/supp-4 Supplemental Information 4 The switching point selected for PtW does not affect bacterial coexistence or phage infection persistence. Sensitivity analyses for the switching point a) at a lysis rate of 0.165 [h^−1^] b) at a lysis rate of 0.0033 [h^−1^] c) at a lysis rate of 0.001 [h^−1^]. The number of coexisting species is shown for increased values of the switching point over a constant and fluctuating resource supply (T = 30 days; a = 0.9). The number of persisting states is given with a color gradient from no state persist (black) to all states—slow and fast growing bacteria, as well as their associated phage and infected bacteria—can persist (yellow). Bifurcation diagrams show the population dynamics for the three switching points.

10.7717/peerj.12194/supp-5 Supplemental Information 5 A variation of the parameters s and SH in Equation 7 (PtL) revealed only marginal effects on population dynamics. Sensitivity analyses of A) the lysis rate s varied from 0.1 to 1 [h^−1^] (SH=4*10^7^). B) the half-saturation density SH varied from 1*10^7^ to 5*10^7^ (s=0.33 [h^−1^]). A bifurcation diagram shows the population dynamics over varying values of s and SH. The number of persisting states is shown for increasing values of s and SH over a constant and fluctuating resource supply (T = 30 days; a = 0.9).

10.7717/peerj.12194/supp-6 Supplemental Information 6 A variation of the parameters s, H and r in Equation 8 (PtW) revealed only marginal effects on population dynamics. Sensitivity analyses of A) the lysis rate s varied from 0.1 to 1 [h^−1^] (H = 1, r = 0.5). B) the parameter H varied from 0.1 to 2 (r = 0.5, s = 0.33 [h^−1^]) C) the parameter r varied from 0.1 to 1 (s = 0.33 [h^−1^], H = 1). A bifurcation diagram shows the population dynamics over varying values of s, H and r. The number of persisting states is shown for increasing values of s, H and r over a constant and fluctuating resource supply (T = 30 days; a = 0.9).

10.7717/peerj.12194/supp-7 Supplemental Information 7 The viral shunt shifts population dynamics to lower resource concentrations for all phage strategies. Bifurcation diagrams of the phage-bacteria models are shown without (left) and with the inclusion of the viral shunt (right) over an increasing resource supply. Equal parameters and initial values were chosen for all simulations (Manuscript Table 1), with a metabolic scaling constant y_1_ = 7.5 for fast and y_2_ = 4 for slow-growing bacteria. Upper graphs show fast growing bacteria, bottom figures the slow growing bacteria and the associated infection. Color code according to Fig. 1 in the manuscript.

10.7717/peerj.12194/supp-8 Supplemental Information 8 Phage infections and slow growing bacteria are more affected by resource fluctuations. The presence of bacteria and their associated infection (infected bacteria and phages) are shown for the lytic infection at varying resource amplitudes or periods over resource supply. Colors represent if bacteria or their infection is present (yellow) or extinct (black). Row 1: Amplitude is given as the percentage of the mean resource supply of 1/24 [h^−1^]. The period was set to 30 days of one resource turnover. Row 2: Period is stated in turnover days, increasing on a logarithmic scale. The amplitude was set to 0.9.

10.7717/peerj.12194/supp-9 Supplemental Information 9 Phage infections and slow growing bacteria are more affected by resource fluctuations. The presence of bacteria and their associated infection (infected bacteria and phages) are shown for PtW at varying resource amplitudes or periods over resource supply. Colors represent if bacteria or their infection is present (yellow) or extinct (black). Row 1: Amplitude is given as the percentage of the mean resource supply of 1/24 [hr^-1^]. The period was set to 30 days of one resource turnover. Row 2: Period is stated in turnover days, increasing on a logarithmic scale. The amplitude was set to 0.9.

10.7717/peerj.12194/supp-10 Supplemental Information 10 Phage infections and slow growing bacteria are more affected by resource fluctuations. The presence of bacteria and their associated infection (infected bacteria and phages) are shown for PtL at varying resource amplitudes or periods over resource supply. Colors represent if bacteria or their infection is present (yellow) or extinct (black). Row 1: Amplitude is given as the percentage of the mean resource supply of 1/24 [h^−1^]. The period was set to 30 days of one resource turnover. Row 2: Period is stated in turnover days, increasing on a logarithmic scale. The amplitude was set to 0.9.

## Additional Information and Declarations

### Data Availability

The following information was supplied regarding data availability: All code files are available at GitHub: https://github.com/Es-Vo-26/Phage-Infection-Strategies.
