# Supplementary material for: Phage strategies facilitate bacterial coexistence under environmental variability
Source: PeerJ. 2021 Nov 4;9:e12194. doi: 10.7717/peerj.12194 (PMC8572521; doi:10.7717/peerj.12194)
Supplement: Supplemental Information 3 — Sensitivity analyses for the switching point a) at a lysis rate of 0.001 [h−1] b) at a lysis rate of 0.0033 [h−1] c) at a lysis rate of 0.165 [h−1]. The number of coexisting species is shown for increased values of the switching point over a constant and fluctuating resource supply (T = 30 days; a = 0.9). The number of persisting states is given with a color gradient from no state persist (black) to all states – slow and fast growing bacteria, as well as their associated phage and infected bacteria—can persist (yellow). Bifurcation diagrams show the population dynamics for the three switching points. [file peerj-09-12194-s003.pdf]

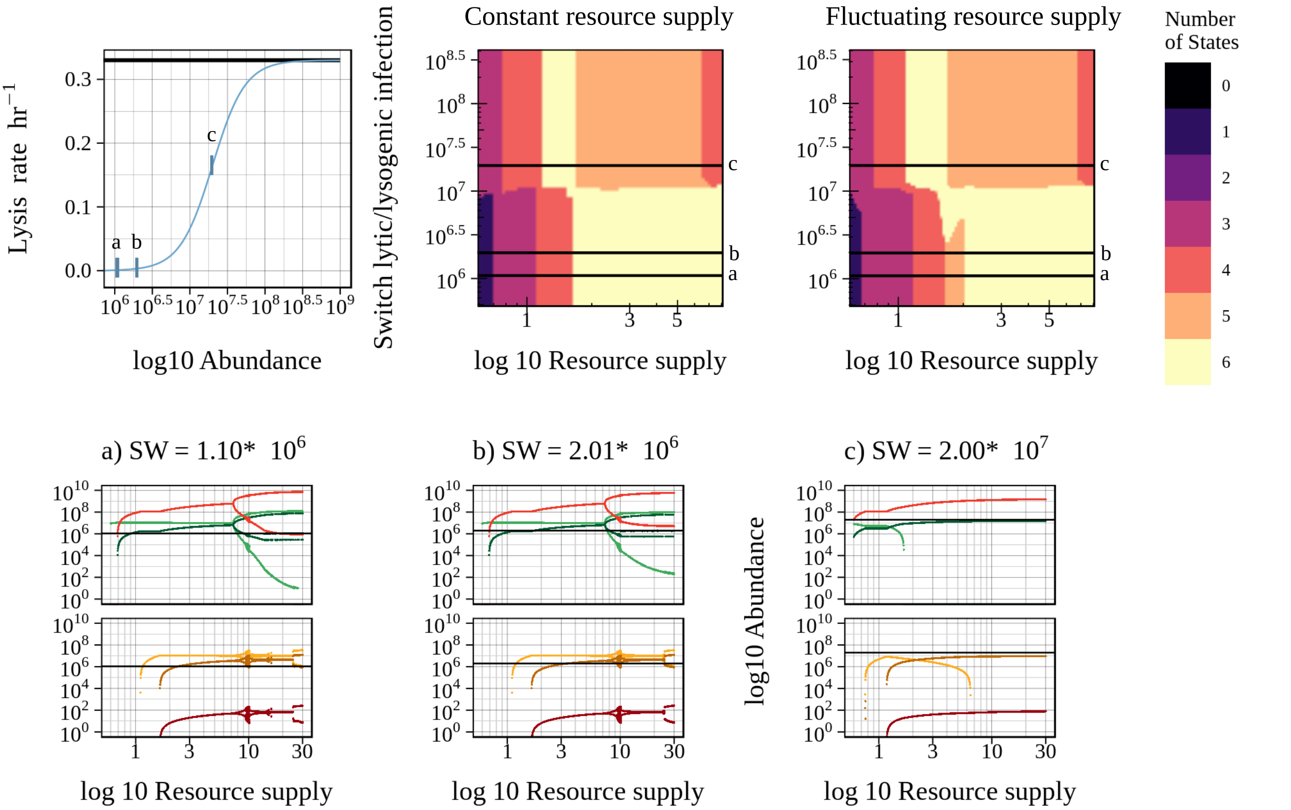

Lysis rate hr^{-1}
log10 Abundance
a
b
c
Switch lytic/lysogenic infection
Constant resource supply
Fluctuating resource supply
log 10 Resource supply
Number of States
0
1
2
3
4
5
6
a) SW = 1.10* 10^6
b) SW = 2.01* 10^6
c) SW = 2.00* 10^7
log10 Abundance
log 10 Resource supply
