# Supplementary material for: Phage strategies facilitate bacterial coexistence under environmental variability
Source: PeerJ. 2021 Nov 4;9:e12194. doi: 10.7717/peerj.12194 (PMC8572521; doi:10.7717/peerj.12194)
Supplement: Supplemental Information 4 — Sensitivity analyses for the switching point a) at a lysis rate of 0.165 [h−1] b) at a lysis rate of 0.0033 [h−1] c) at a lysis rate of 0.001 [h−1]. The number of coexisting species is shown for increased values of the switching point over a constant and fluctuating resource supply (T = 30 days; a = 0.9). The number of persisting states is given with a color gradient from no state persist (black) to all states—slow and fast growing bacteria, as well as their associated phage and infected bacteria—can persist (yellow). Bifurcation diagrams show the population dynamics for the three switching points. [file peerj-09-12194-s004.pdf]

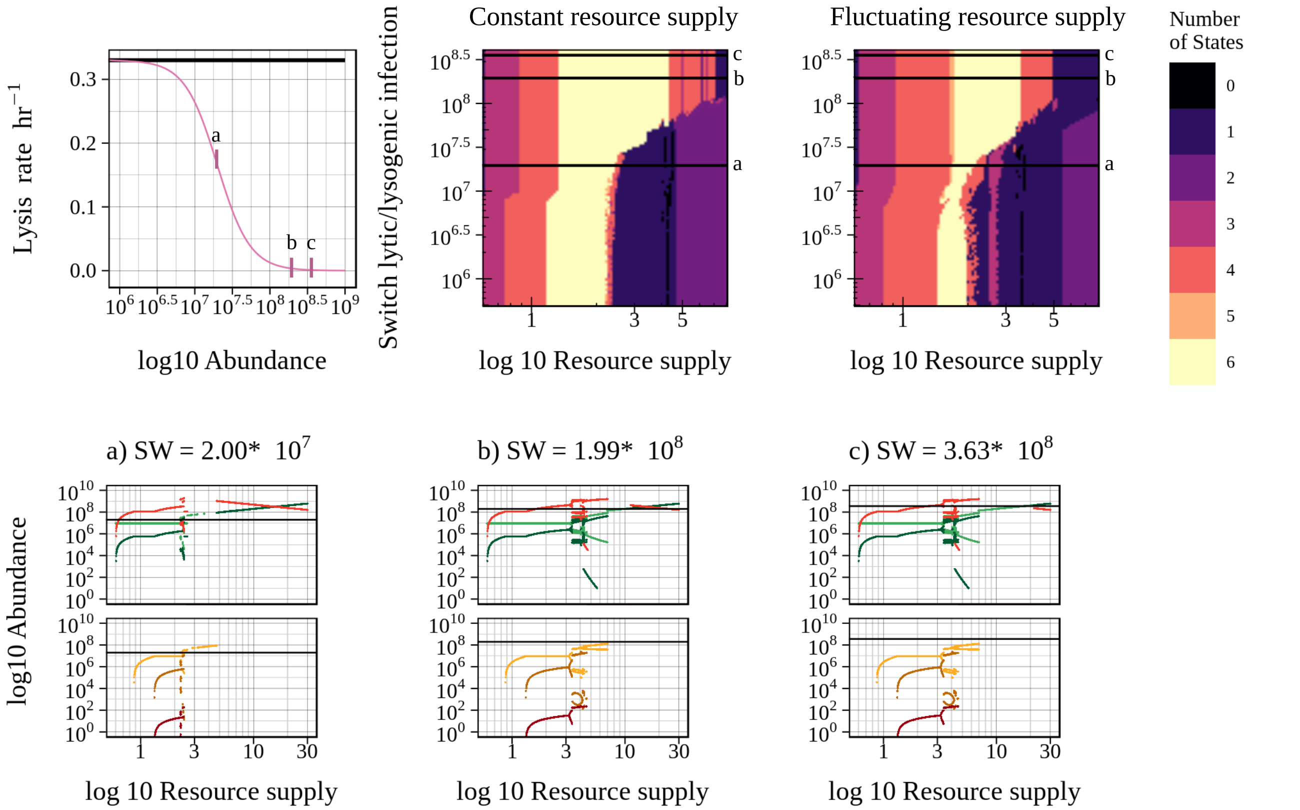

Lysis rate hr^-1
log10 Abundance
Switch lytic/lysogenic infection
Constant resource supply
Fluctuating resource supply
log 10 Resource supply
Number of States
0
1
2
3
4
5
6
a
b
c
a) SW = 2.00* 10^7
b) SW = 1.99* 10^8
c) SW = 3.63* 10^8
log10 Abundance
log 10 Resource supply
