# Supplementary figures and images for: Phage strategies facilitate bacterial coexistence under environmental variability
Source: PeerJ. 2021 Nov 4;9:e12194. doi: 10.7717/peerj.12194 (PMC8572521; doi:10.7717/peerj.12194)

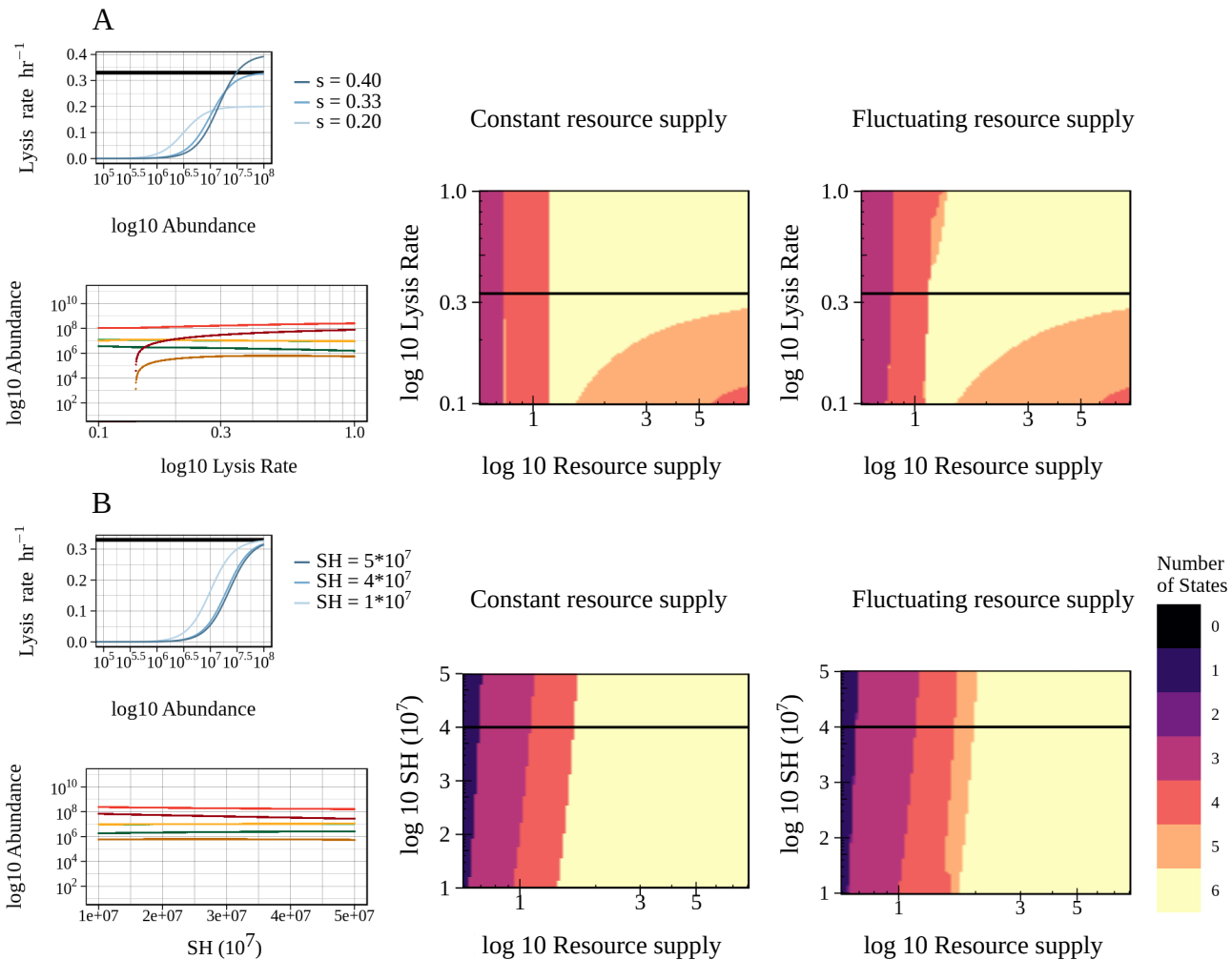

Supplement: Supplemental Information 5 — Sensitivity analyses of A) the lysis rate s varied from 0.1 to 1 [h−1] (SH=4*107). B) the half-saturation density SH varied from 1*107 to 5*107 (s=0.33 [h−1]). A bifurcation diagram shows the population dynamics over varying values of s and SH. The number of persisting states is shown for increasing values of s and SH over a constant and fluctuating resource supply (T = 30 days; a = 0.9). [file peerj-09-12194-s005.pdf]

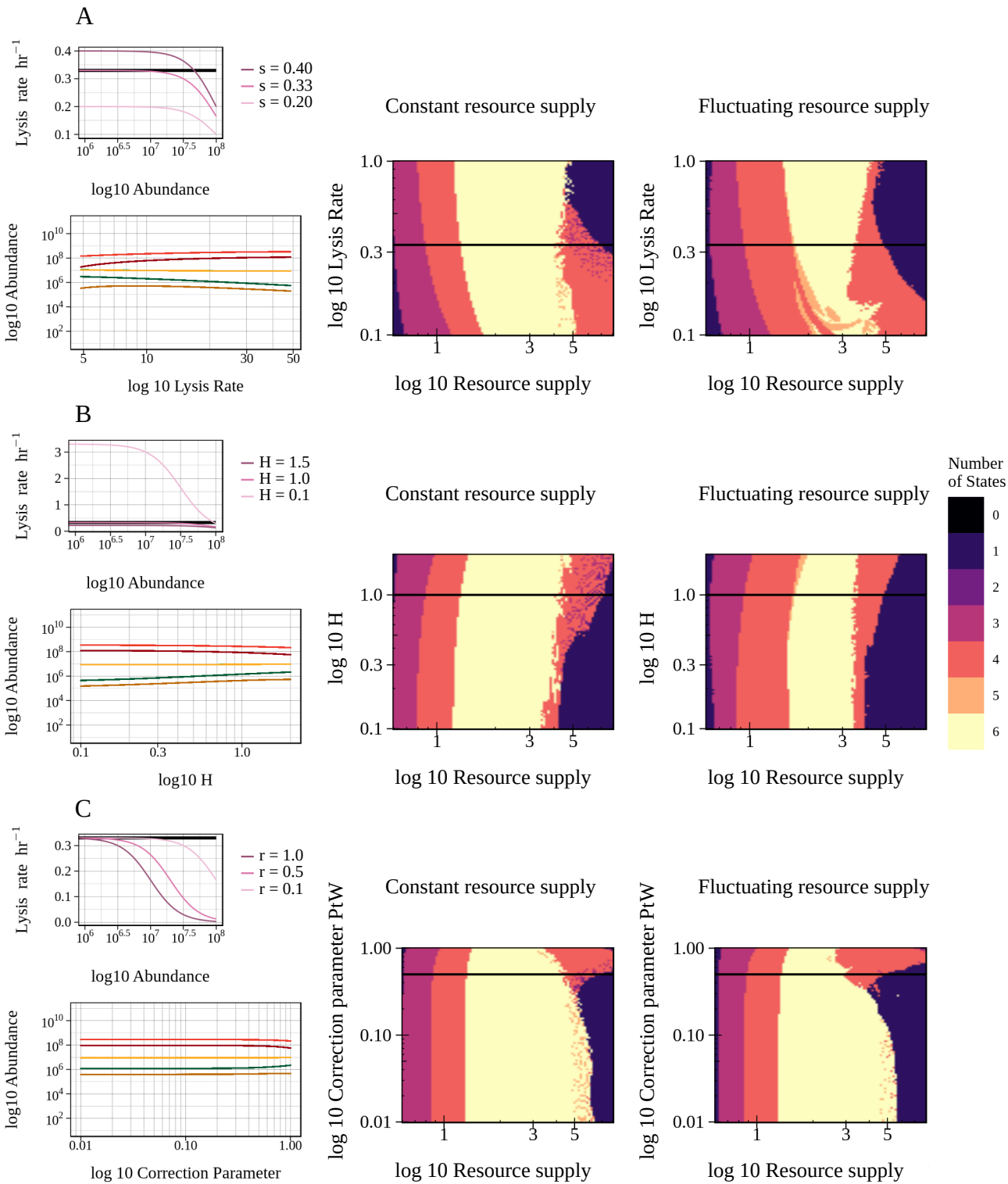

Supplement: Supplemental Information 6 — Sensitivity analyses of A) the lysis rate s varied from 0.1 to 1 [h−1] (H = 1, r = 0.5). B) the parameter H varied from 0.1 to 2 (r = 0.5, s = 0.33 [h−1]) C) the parameter r varied from 0.1 to 1 (s = 0.33 [h−1], H = 1). A bifurcation diagram shows the population dynamics over varying values of s, H and r. The number of persisting states is shown for increasing values of s, H and r over a constant and fluctuating resource supply (T = 30 days; a = 0.9). [file peerj-09-12194-s006.pdf]

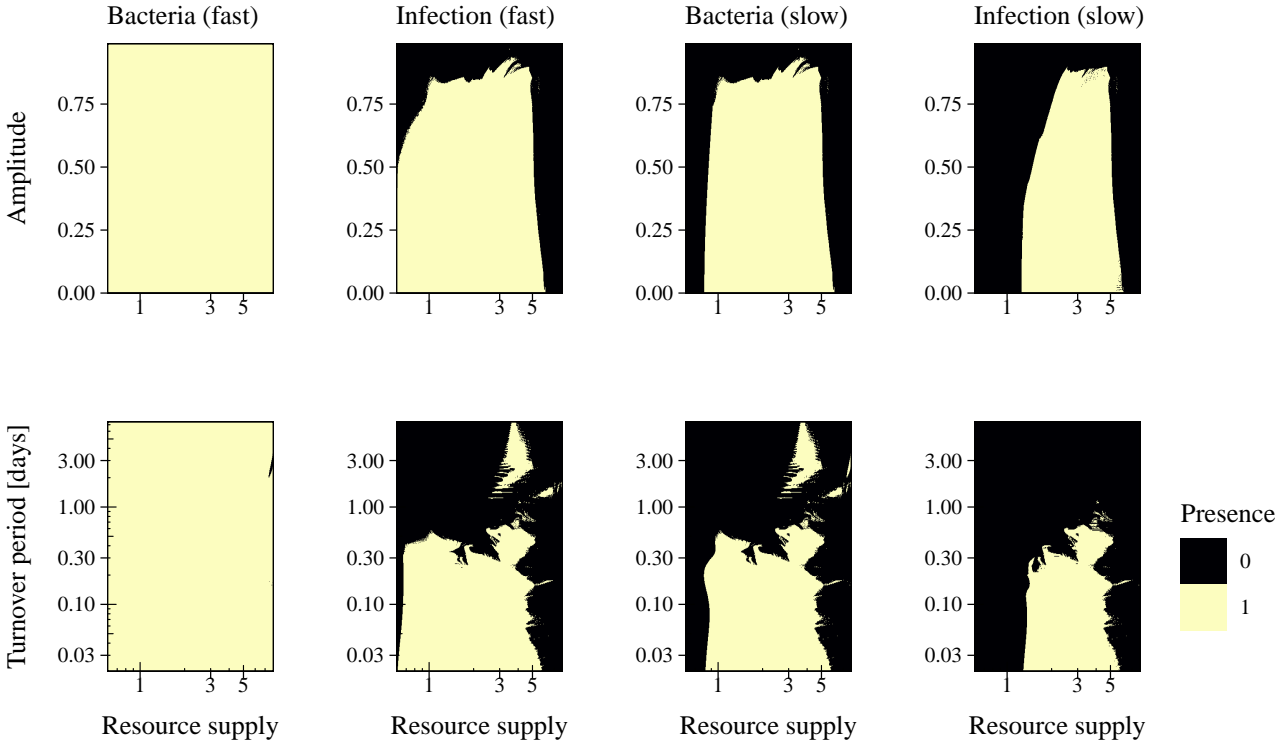

Supplement: Supplemental Information 8 — The presence of bacteria and their associated infection (infected bacteria and phages) are shown for the lytic infection at varying resource amplitudes or periods over resource supply. Colors represent if bacteria or their infection is present (yellow) or extinct (black). Row 1: Amplitude is given as the percentage of the mean resource supply of 1/24 [h−1]. The period was set to 30 days of one resource turnover. Row 2: Period is stated in turnover days, increasing on a logarithmic scale. The amplitude was set to 0.9. [file peerj-09-12194-s008.pdf]

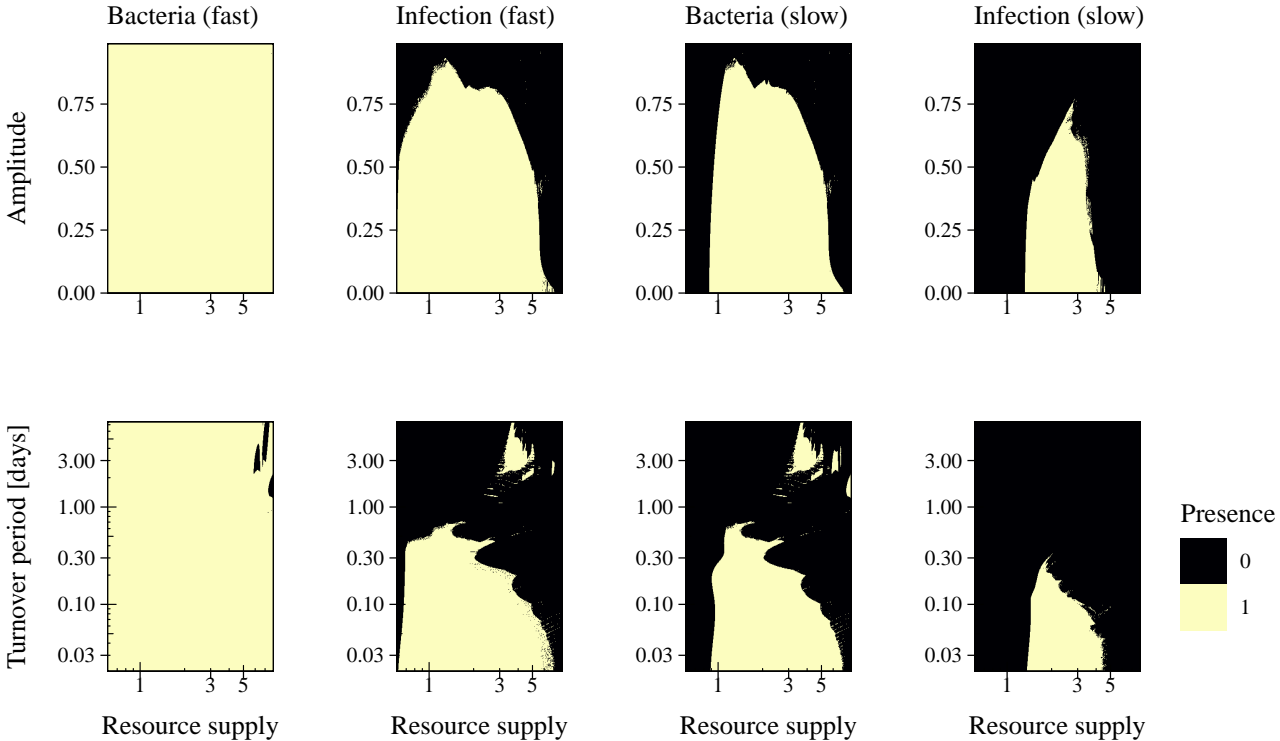

Supplement: Supplemental Information 9 — The presence of bacteria and their associated infection (infected bacteria and phages) are shown for PtW at varying resource amplitudes or periods over resource supply. Colors represent if bacteria or their infection is present (yellow) or extinct (black). Row 1: Amplitude is given as the percentage of the mean resource supply of 1/24 [hr-1]. The period was set to 30 days of one resource turnover. Row 2: Period is stated in turnover days, increasing on a logarithmic scale. The amplitude was set to 0.9. [file peerj-09-12194-s009.pdf]

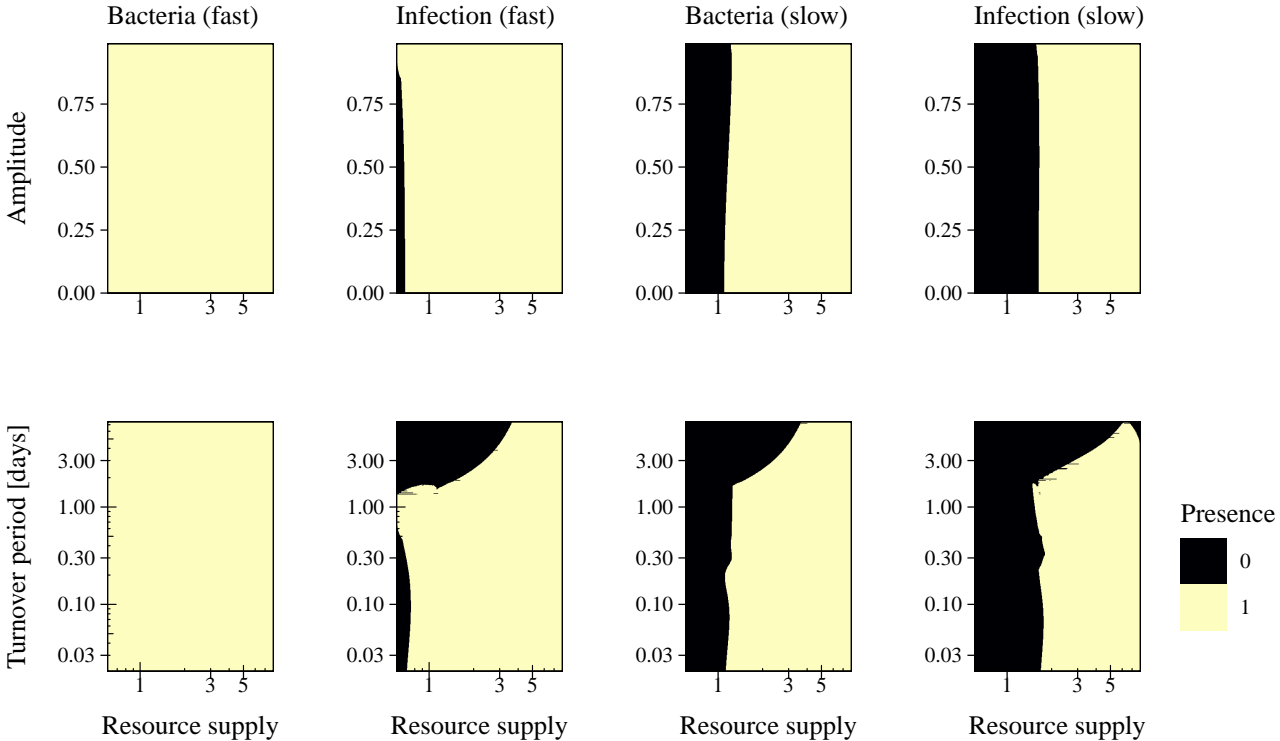

Supplement: Supplemental Information 10 — The presence of bacteria and their associated infection (infected bacteria and phages) are shown for PtL at varying resource amplitudes or periods over resource supply. Colors represent if bacteria or their infection is present (yellow) or extinct (black). Row 1: Amplitude is given as the percentage of the mean resource supply of 1/24 [h−1]. The period was set to 30 days of one resource turnover. Row 2: Period is stated in turnover days, increasing on a logarithmic scale. The amplitude was set to 0.9. [file peerj-09-12194-s010.pdf]
