# Supplementary material for: Phage strategies facilitate bacterial coexistence under environmental variability
Source: PeerJ. 2021 Nov 4;9:e12194. doi: 10.7717/peerj.12194 (PMC8572521; doi:10.7717/peerj.12194)
Supplement: Supplemental Information 7 — Bifurcation diagrams of the phage-bacteria models are shown without (left) and with the inclusion of the viral shunt (right) over an increasing resource supply. Equal parameters and initial values were chosen for all simulations (Manuscript Table 1), with a metabolic scaling constant y1 = 7.5 for fast and y2 = 4 for slow-growing bacteria. Upper graphs show fast growing bacteria, bottom figures the slow growing bacteria and the associated infection. Color code according to Fig. 1 in the manuscript. [file peerj-09-12194-s007.pdf]

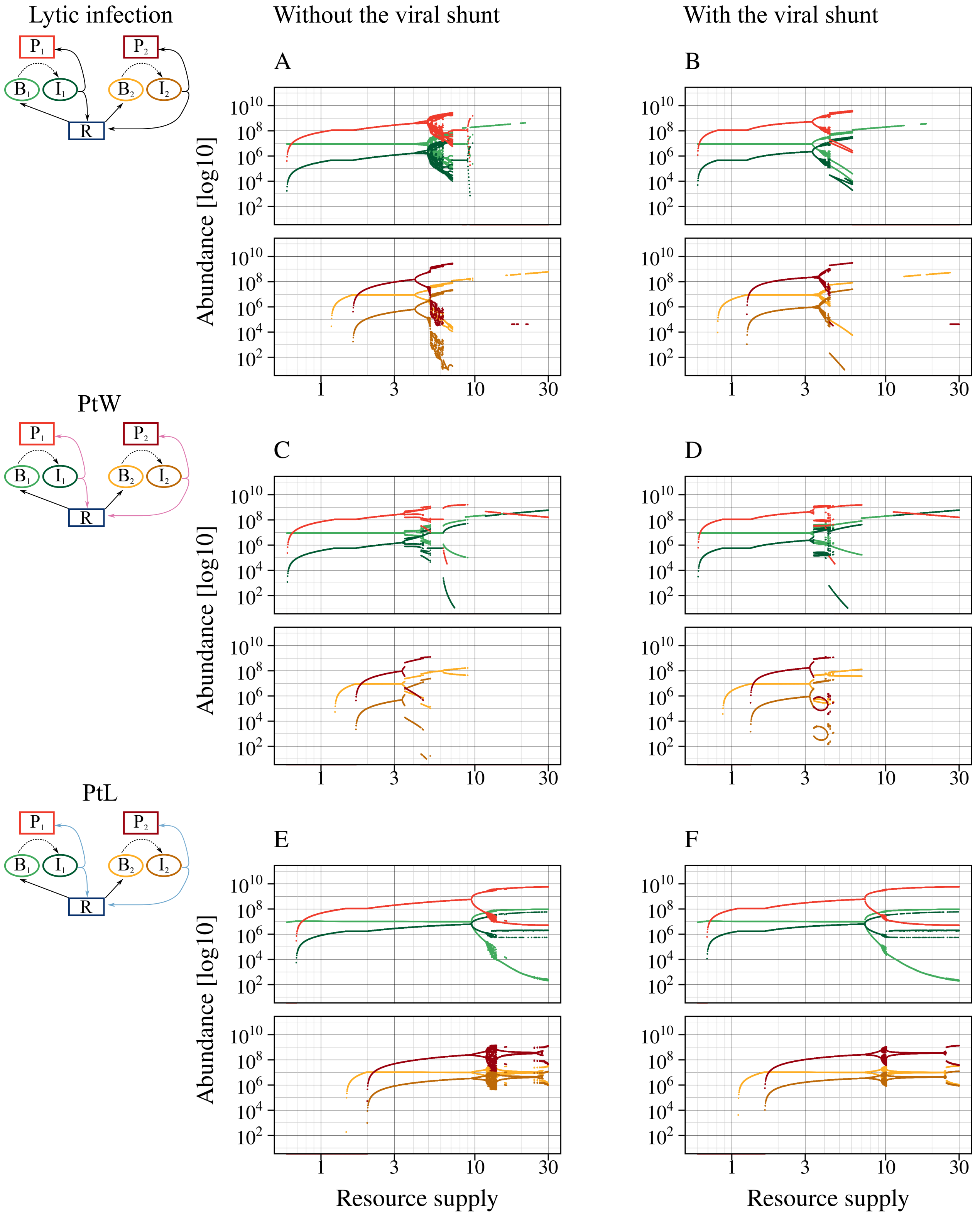
Lytic infection
Without the viral shunt
With the viral shunt
A
B
PtW
C
D
PtL
E
F
Abundance [log10]
Resource supply
