# Supplementary material for: Phage strategies facilitate bacterial coexistence under environmental variability
Source: PeerJ. 2021 Nov 4;9:e12194. doi: 10.7717/peerj.12194 (PMC8572521; doi:10.7717/peerj.12194)
Supplement: Supplementary file 7 [file peerj-09-12194-s001.odt]

Supplement Material

# Methods

Normalized biovolumes of phage and bacteria

We transformed abundances and densities in our model into biovolume and normalized the values by the half-saturation density, NH (Supplementary Table 1). For a transformation into biovolume, we used the biovolume of heterotrophic prokaryotes for bacteria (0.523 µm3) (Bengtsson 1989) and the mean value of 7 different phage radii (1.641*10-4 µm3) (Jover et al. 2014). The biovolumes of bacteria and phages were normalized by the half-saturation density, NH= 5.23 * 106 [µm3 mL-1].

We converted the infection rate (i) with the phage biovolume and normalized it with the half-saturation density, NH.

## Sensitivity Analysis

We added a switching point to the temperate phage models, to turn the growth of infected bacteria on or off. The switching point was set to a lysis rate of 0.0033 [hr-1] (Supplementary Figure 1, 2) for both phage strategies.

The phage induction of the temperate phage strategies (PtL and PtW) is simulated by altering the lysis rate. The phage induction was defined by an increasing or decreasing sigmoidal function for PtL and PtW, respectively. We have chosen the parameters to specify these functions in the same host density range. A sensitivity analysis revealed only marginal effects of these parameters (Supplementary Figure 3, 4).

# Results

We used bifurcation diagrams, to analyze the effect of the viral shunt (Supplementary Figure 5). We examined bacterial coexistence, the persistence of the phage infection, oscillations, and species extinction over a resource supply, N0, systematically varying between 0.6 and 30. The addition of the viral shunt into the model leads to an enrichment of the system (Supplementary Figure 5). As a result, slow and fast growing bacteria were able to coexist at lower resource concentrations. The viral shunt was included in all phage infection mechanisms for further calculations.

We specified the impact of resource fluctuations on population stability by simulating the three phage strategies while a) independently varying relative amplitude a (0 % to 99 %) and nutrient supply N0 (period fixed at p = 30 days); and b) independently varying period (1 day to 1 year) and nutrient supply N0 (relative amplitude fixed at a = 90 %) (Supplementary Figure 6, 7, 8). The presence of slow and fast growing bacteria and their infection (infected bacteria and phages) was plotted for an increasing a) amplitude or b) period over resource supply. The color depicts persistence (yellow) or extinction (black). Our results show that slow growing bacteria and their associated infection goes extinct first, followed by the infection of the fast growing bacteria (Supplementary Figure 6, 7, 8). The persistence of the fast growing bacteria was not affected by resource fluctuations.
