# Supplementary material for: Phage strategies facilitate bacterial coexistence under environmental variability
Source: PeerJ. 2021 Nov 4;9:e12194. doi: 10.7717/peerj.12194 (PMC8572521; doi:10.7717/peerj.12194)
Supplement: Supplementary file 8 [file peerj-09-12194-s002.odt]

| Name | Value | Unit | Biovolume | Unit | norm. biovolume | Unit |
| --- | --- | --- | --- | --- | --- | --- |
| Bacteria (B) | 8.30 * 106 | particle mL-1 | 4.34 * 106 | µm3 mL-1 | 8.30 * 10-1 |  |
| Infected bacteria (Bi) | 1.70 * 106 | particle mL-1 | 8.90 * 105 | µm3 mL-1 | 1.70 * 10-1 |  |
| Phages (P) | 1.00 * 107 | particle mL-1 | 1.64 * 103 | µm3 mL-1 | 3.10 * 10-4 |  |
| Half-saturation density (NH) | 1.00 * 107 | particle mL-1 | 5.23 * 106 | µm3 mL-1 | 1.00 |  |
| Switch point PtW | 1.99 * 108 | particles mL-1 | 1.04 * 108 | µm3 mL-1 | 19.8997500 |  |
| Switch point PtL | 2.01 * 106 | particles mL-1 | 1.05 * 106 | µm3 mL-1 | 0.2010076 |  |
| Half-saturation density (SH) | 4.00 * 107 | particles mL-1 | 2.09 * 107 | µm3 mL-1 | 4.00 |  |
| Infection rate (i) | 1.64 * 10-10 | mL hr-1 | 9.99 * 10-7 | mL µm-3 hr-1 | 5.23 | hr-1 |
